# Correction: Understanding the Underlying Mechanism of HA-Subtyping in the Level of Physic-Chemical Characteristics of Protein

**DOI:** 10.1371/journal.pone.0099921

**Published:** 2014-06-04

**Authors:** The PLOS ONE Staff

## Notice of Republication

This article was republished on May 20, 2014, due to an error in the title. The citation has been updated to match the corrected title. Please download this article again to view the corrected title and citation. The originally published, uncorrected article and the republished, corrected article are provided here for reference.

## Supporting Information

File S1 **Originally published, uncorrected article.**
(PDF)

File S2 **Republished, corrected article.**
(PDF)
