# Supplementary figures and images for: Understanding the Underlying Mechanism of HA-Subtyping in the Level of Physic-Chemical Characteristics of Protein
Source: PLoS One. 2014 May 8;9(5):e96984. doi: 10.1371/journal.pone.0096984 (PMC4014573; doi:10.1371/journal.pone.0096984)

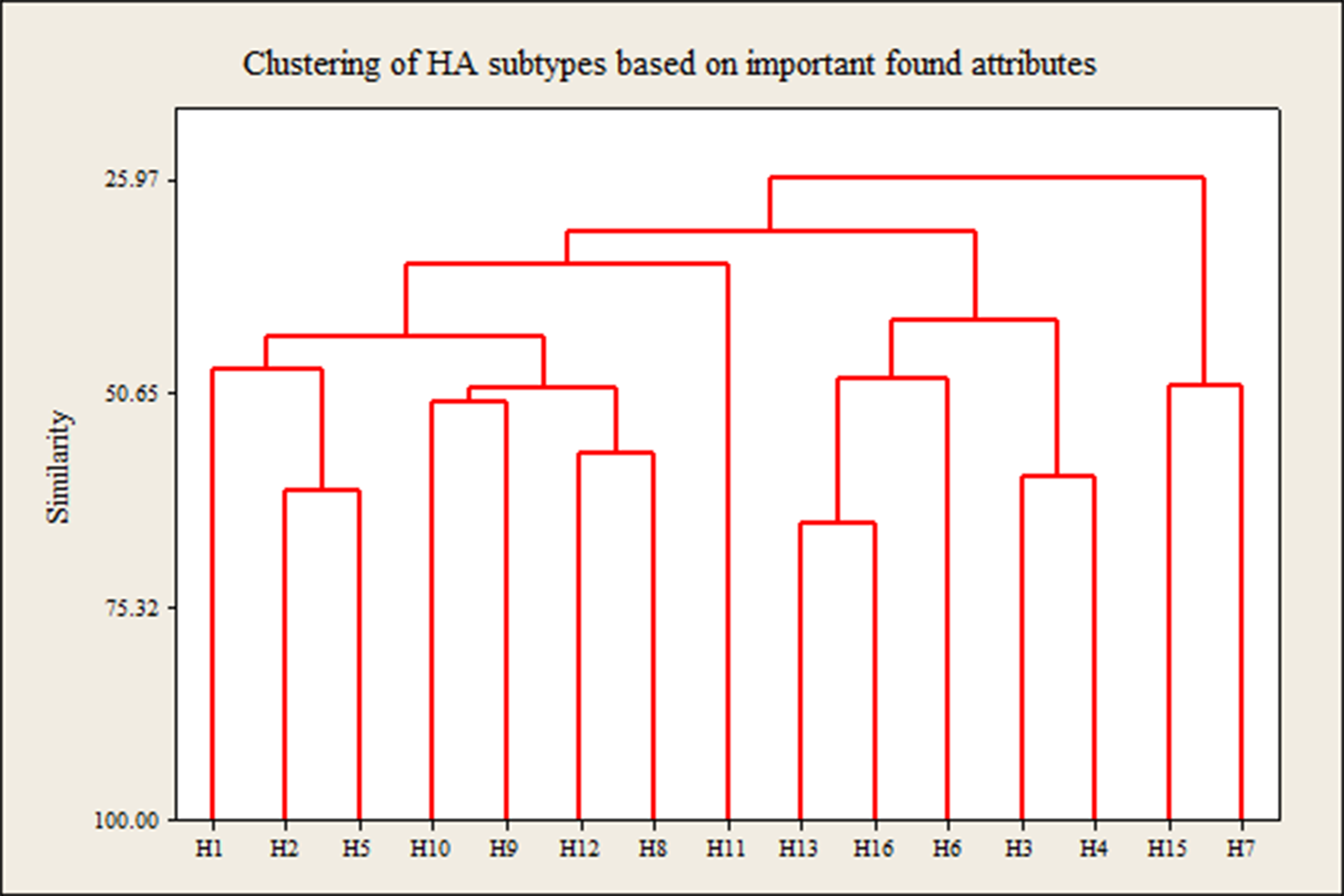

Supplement: Figure S1 — Clustering of proteins based on the 16 important features found in this study. The key discovered features (obtained by attribute weighting and decision tree models) are highly accurate in prediction and separation of HA subtypes and reinforces the high capability of discovered features as predictors of HA subtyping. The features include: Non-reduced cysteines Ext, Count of Isoleucine, Freq of Cysteine, Freq of Aspartic Acid, Freq of Glutamic Acid, Freq of Glutamine, Freq of Arginine, Freq of Tyrosine, Percentage of Histidine, Percentage of Methionin, Percentage of Tryptophan, Percentage of Tyrosine, Count of Phe-Met, Count of Asn-Met, Freq of Pro-Gly, and Freq of Trp-Leu. (TIF) [file pone.0096984.s001.tif]
