# Supplementary material for: Understanding the Underlying Mechanism of HA-Subtyping in the Level of Physic-Chemical Characteristics of Protein
Source: PLoS One. 2014 May 8;9(5):e96984. doi: 10.1371/journal.pone.0096984 (PMC4014573; doi:10.1371/journal.pone.0096984)
Supplement: Table S1 — The original cleaned protein feature data set for HA sequences (Fcdb). This database contains 7338 protein sequences and 893 protein features. Table S1 is shared by “Googledrive” at the following link: https://drive.google.com/file/d/0B2Npj-saFbgeNjhwRTJubFRJdFk/edit?usp=sharing. (DOCX) [file pone.0096984.s002.docx]

**Supplementary Table S1.** The original cleaned protein feature data set for HA sequences (Fcdb). This database contains 7338 protein sequences and 893 protein features. Table S1 is shared by “Googledrive” at the following link:

<https://drive.google.com/file/d/0B2Npj-saFbgeNjhwRTJubFRJdFk/edit?usp=sharing>
