# Supplementary material for: Understanding the Underlying Mechanism of HA-Subtyping in the Level of Physic-Chemical Characteristics of Protein
Source: PLoS One. 2014 May 8;9(5):e96984. doi: 10.1371/journal.pone.0096984 (PMC4014573; doi:10.1371/journal.pone.0096984)
Supplement: Table S3 — Univariate Analysis of Variance (ANOVA) and Multivariate Analyses of Variance (MANOVA) of the effects of important amino acid attributes (selected by attribute weighting algorithms) and some of the important dipeptides (selected by decision tree) models on determining HA subtypes (H1-H16). (DOCX) [file pone.0096984.s004.docx]

**Table S3. Univariate Analysis of Variance (ANOVA) and Multivariate Analyses of Variance (MANOVA) of the effects of important amino acid attributes (selected by attribute weighting algorithms) and some of the important dipeptides (selected by decision tree) models on determining HA subtypes (H1-H16).**

**Part A - Univariate Analysis**

Analysis of Variance for Non-reduced cysteines Extinction Cofficient

Source DF Seq SS Adj SS Adj MS F P

Type 14 3.52842E+11 3.52842E+11 25202977014 3732.22 0.000

Error 7323 49450808484 49450808484 6752807

Total 7337 4.02292E+11

S = 2598.62 R-Sq = 87.71% R-Sq(adj) = 87.68%

------------------------------------------------------------------------

Analysis of Variance for Count of Isoleucine (I)

Source DF Seq SS Adj SS Adj MS F P

Type 14 203755 203755 14554 3750.54 0.000

Error 7323 28417 28417 4

Total 7337 232171

S = 1.96989 R-Sq = 87.76% R-Sq(adj) = 87.74%

-----------------------------------------------------------------------------

Analysis of Variance for Freq of Cysteine (C)

Source DF Seq SS Adj SS Adj MS F P

Type 14 0.0355750 0.0355750 0.0025411 7800.03 0.000

Error 7323 0.0023857 0.0023857 0.0000003

Total 7337 0.0379607

S = 0.000570769 R-Sq = 93.72% R-Sq(adj) = 93.70%

----------------------------------------------------------------------------

Analysis of Variance for Freq of Aspartic Acid (D)

Source DF Seq SS Adj SS Adj MS F P

Type 14 0.167744 0.167744 0.011982 761.40 0.000

Error 7323 0.115238 0.115238 0.000016

Total 7337 0.282983

S = 0.00396693 R-Sq = 59.28% R-Sq(adj) = 59.20%

----------------------------------------------------------------------------

Analysis of Variance for Freq of Glutamic Acid (E)

Source DF Seq SS Adj SS Adj MS F P

Type 14 0.400826 0.400826 0.028630 2698.98 0.000

Error 7323 0.077682 0.077682 0.000011

Total 7337 0.478508

S = 0.00325698 R-Sq = 83.77% R-Sq(adj) = 83.73%

--------------------------------------------------------------------------------

Analysis of Variance for Freq of Glutamine (Q)

Source DF Seq SS Adj SS Adj MS F P

Type 14 0.429960 0.429960 0.030711 11811.94 0.000

Error 7323 0.019040 0.019040 0.000003

Total 7337 0.449000

S = 0.00161246 R-Sq = 95.76% R-Sq(adj) = 95.75%

-------------------------------------------------------------------------------

Analysis of Variance for Freq of Arginine (R),

Source DF Seq SS Adj SS Adj MS F P

Type 14 0.376558 0.376558 0.026897 4085.75 0.000

Error 7323 0.048208 0.048208 0.000007

Total 7337 0.424766

S = 0.00256576 R-Sq = 88.65% R-Sq(adj) = 88.63%

--------------------------------------------------------------------------------

Analysis of Variance for Freq of Tyrosine (Y),

Source DF Seq SS Adj SS Adj MS F P

Type 14 0.339244 0.339244 0.024232 14304.53 0.000

Error 7323 0.012405 0.012405 0.000002

Total 7337 0.351649

S = 0.00130153 R-Sq = 96.47% R-Sq(adj) = 96.47%

--------------------------------------------------------------------------------

Analysis of Variance for Percentage of Histidine (H)

Source DF Seq SS Adj SS Adj MS F P

Type 14 686.971 686.971 49.069 3595.18 0.000

Error 7323 99.949 99.949 0.014

Total 7337 786.920

S = 0.116827 R-Sq = 87.30% R-Sq(adj) = 87.27%

--------------------------------------------------------------------------------

Analysis of Variance for Percentage of Methionine (M)

Source DF Seq SS Adj SS Adj MS F P

Type 14 1298.416 1298.416 92.744 3080.45 0.000

Error 7323 220.475 220.475 0.030

Total 7337 1518.891

S = 0.173514 R-Sq = 85.48% R-Sq(adj) = 85.46%

--------------------------------------------------------------------------------

Analysis of Variance for Percentage of Tryptophan (W),

Source DF Seq SS Adj SS Adj MS F P

Type 14 214.383 214.383 15.313 3259.07 0.000

Error 7323 34.408 34.408 0.005

Total 7337 248.791

S = 0.0685464 R-Sq = 86.17% R-Sq(adj) = 86.14%

-------------------------------------------------------------------------------

Analysis of Variance for Percentage of Tyrosine (Y),

Source DF Seq SS Adj SS Adj MS F P

Type 14 3314.78 3314.78 236.77 14284.66 0.000

Error 7322 121.36 121.36 0.02

Total 7336 3436.14

S = 0.128744 R-Sq = 96.47% R-Sq(adj) = 96.46%

-------------------------------------------------------------------------------

Analysis of Variance for Count of Phe-Met

Source DF Seq SS Adj SS Adj MS F P

Type 14 102.3994 102.3994 7.3142 745.52 0.000

Error 7323 71.8453 71.8453 0.0098

Total 7337 174.2448

S = 0.0990501 R-Sq = 58.77% R-Sq(adj) = 58.69%

-------------------------------------------------------------------------------

Analysis of Variance for Count of Asn-Met,

Source DF Seq SS Adj SS Adj MS F P

Type 14 1047.313 1047.313 74.808 4303.71 0.000

Error 7323 127.290 127.290 0.017

Total 7337 1174.603

S = 0.131842 R-Sq = 89.16% R-Sq(adj) = 89.14%

--------------------------------------------------------------------------------

Analysis of Variance for Freq of Pro-Gly,

Source DF Seq SS Adj SS Adj MS F P

Type 14 0.0040267 0.0040267 0.0002876 681.09 0.000

Error 7323 0.0030925 0.0030925 0.0000004

Total 7337 0.0071192

S = 0.000649841 R-Sq = 56.56% R-Sq(adj) = 56.48%

--------------------------------------------------------------------------------

Analysis of Variance for Freq of Trp-Leu,

Source DF Seq SS Adj SS Adj MS F P

Type 14 0.0053261 0.0053261 0.0003804 2933.38 0.000

Error 7323 0.0009497 0.0009497 0.0000001

Total 7337 0.0062758

S = 0.000360127 R-Sq = 84.87% R-Sq(adj) = 84.84%

--------------------------------------------------------------------------------

**Part B - Multivariate Analysis (MANOVA)**

(The simultaneous effect of Non-reduced cysteines Extinction Cofficient,

Count of Isoleucine (I), Freq of Cysteine (C), Freq of Aspartic Acid (D),

Freq of Glutamic Acid (E), Freq of Glutamine (Q), Freq of Arginine (R),

Freq of Tyrosine (Y),Percentage of Histidine (H, Percentage of Methionine (M),

Percentage of Tryptophan (W),Percentage of Tyrosine (Y), Count of Phe-Met, Count of Asn-Met,Freq of Pro-Gly,and Freq of Trp-Leu on differentiation of H subtypes, H1-H16, was analysed)

MANOVA for HA subtype

Criterion Statistic Approx F Num Denom P

Wilks' 0.00000 1657.242 224 77445 0.000

Lawley-Hotelling 165.59586 5400.453 224 102272 0.000

Pillai's 7.24488 490.670 224 102480 0.000

Roy's 101.82111
