# Supplementary material for: Correction: Understanding the Underlying Mechanism of HA-Subtyping in the Level of Physic-Chemical Characteristics of Protein
Source: PLoS One. 2014 Jun 4;9(6):e99921. doi: 10.1371/journal.pone.0099921 (PMC4045987; doi:10.1371/journal.pone.0099921)
Supplement: File S2 — Republished, corrected article. (PDF) [file pone.0099921.s002.pdf]

OPEN ACCESS Freely available online

PLOS | ONE

Understanding the Underlying Mechanism of HA-Subtyping in the Level of Physic-Chemical Characteristics of Protein

Mansour Ebrahimi[1], Parisa Aghagolzadeh[2], Narges Shamabadi[1], Ahmad Tahmasebi[3], Mohammed Alsharifi[4], David L. Adelson[4], Farhid Hemmatzadeh[5]*, Esmaeil Ebrahimie[4]*

1 Department of Biology, School of Basic Sciences, University of Qom, Qom, Iran, **2** Department of Nephrology, Hypertension, and Clinical Pharmacology, University of Bern, Bern, Switzerland, **3** Institute of Biotechnology, Shiraz University, Shiraz, Iran, **4** School of Molecular and Biomedical Science, The University of Adelaide, Adelaide, Australia, **5** School of Animal and Veterinary Science, The University of Adelaide, Adelaide, Australia

Abstract

The evolution of the influenza A virus to increase its host range is a major concern worldwide. Molecular mechanisms of increasing host range are largely unknown. Influenza surface proteins play determining roles in reorganization of host-sialic acid receptors and host range. In an attempt to uncover the physic-chemical attributes which govern HA subtyping, we performed a large scale functional analysis of over 7000 sequences of 16 different HA subtypes. Large number (896) of physic-chemical protein characteristics were calculated for each HA sequence. Then, 10 different attribute weighting algorithms were used to find the key characteristics distinguishing HA subtypes. Furthermore, to discover machine leaning models which can predict HA subtypes, various Decision Tree, Support Vector Machine, Naïve Bayes, and Neural Network models were trained on calculated protein characteristics dataset as well as 10 trimmed datasets generated by attribute weighting algorithms. The prediction accuracies of the machine learning methods were evaluated by 10-fold cross validation. The results highlighted the frequency of Gln (selected by 80% of attribute weighting algorithms), percentage/frequency of Tyr, percentage of Cys, and frequencies of Try and Glu (selected by 70% of attribute weighting algorithms) as the key features that are associated with HA subtyping. *Random Forest* tree induction algorithm and *RBF kernel* function of *SVM* (scaled by grid search) showed high accuracy of 98% in clustering and predicting HA subtypes based on protein attributes. Decision tree models were successful in monitoring the short mutation/reassortment paths by which influenza virus can gain the key protein structure of another HA subtype and increase its host range in a short period of time with less energy consumption. Extracting and mining a large number of amino acid attributes of HA subtypes of influenza A virus through supervised algorithms represent a new avenue for understanding and predicting possible future structure of influenza pandemics.

Citation: Ebrahimi M, Aghagolzadeh P, Shamabadi N, Tahmasebi A, Alsharifi M, et al. (2014) Understanding the Underlying Mechanism of HA-Subtyping in the Level of Physic-Chemical Characteristics of Protein. PLoS ONE 9(5): e96984. doi:10.1371/journal.pone.0096984

Editor: Stephen Mark Tompkins, University of Georgia, United States of America

Received April 1, 2013; **Accepted** April 7, 2014; **Published** May 8, 2014

Copyright: © 2014 Ebrahimi et al. This is an open-access article distributed under the terms of the Creative Commons Attribution License, which permits unrestricted use, distribution, and reproduction in any medium, provided the original author and source are credited.

Funding: This study has been granted through Australian Centre for International Agricultural Research, ACIAR (Project number: AH2006-050). The funders had no role in study design, data collection and analysis, decision to publish, or preparation of the manuscript.

Competing Interests: The authors have declared that no competing interests exist.

* E-mail: farhid.hemmatzadeh@adelaide.edu.au (FH); esmaeil.ebrahimie@adelaide.edu.au (EE)

These authors contributed equally to this work.

Introduction

Increasing host range of Influenza A virus is a major concern worldwide [1,2]. Avian H5N1 influenza has been infecting humans zoonotically since 1997, resulting in a high mortality rate and there were fears that it might cause the first pandemic of the 21st century. Recent 2013 human infection with a novel avian-origin Influenza A (H7N9) virus with pandemic potential has caused significant concern with more than 130 human cases of severe infection in China and 43 fatalities [3–6]. It should be noted that due to non-existence of previous host immunity, emergence of new broad host range of influenza strain with the ability of human-to-human transmission could result in a pandemic with millions of fatalities [7,8].

Influenza A virus evolve in a complex manner with high frequency of genomic alteration, mainly because of: (1) its intrinsic segmented RNA genomic structure facilitating high frequency of genetic reassortment and antigenic drift [9], (2) circulating different subtypes of HA and NA as available genetic materials for genetic alteration and pandemic induction [10], and (3) availability of hosting environments such as swine as a shelter against humans or birds vaccines and as a mixing vessel for generating reassorted viruses [10,11].

Surface glycoproteins, haemagglutinin (HA) and neuraminidase (NA), determine subtypes of influenza A virus of which 16 HA subtypes and 9 NA subtypes have been identified [12–14]. The HA and NA genes are extremely variable in sequence, and less than 30% of the amino acids are conserved among all subtypes. Host range/specificity of influenza differs due to its surface proteins. HA is the key responsible of viral infectivity and specificity [15,16]. When virus releases from host cells, NA

catalyzes the cleavage of a-ketosidic linkage between a terminal sialic acid and an adjacent D-galactose [9].

Alteration of viral surface proteins to recognize a range of host receptors is the strategy by which influenza increases its host range [2,14]. Interestingly, despite its complexity, recent studies shows that a few amino acid substitutions (4–5) has the potential of altering A/Indonesia/5/2005 avian A/H5N1 and A/Vietnam/1203/2004 A/H5N1 to be transmissible between ferrets via respiratory droplets [2,17–19]. Converting nonlethal H5N1 influenza virus isolated from a human to a lethal virus in mice happened by a single amino acid substitution from glutamic acid to lysine at the position 627 of the PB2 protein [20]. While the origin isolate solely replicates in respiratory organs, the lethal isolate has the ability to replicate in a variety of organs and produce systemic infection [20]. It seems that amino acid profiling of surface influenza proteins has the potential to monitor the host specificity.

Allignment based methods including similarity search (BLAST), sequence alignment, and clustering have the ability to cluster HA sequences with high accuracy. However, these unsupervised models only work with one feature of sequence similarity and do not provide knowledge in deeper levels of functional/structural protein architecture. In contrast, application of supervised models provides the possibility of large scale rule discovery (associated to label variable) where this opportunity is restricted in common unsupervised (clustering) methods. The extracted rules and knowledge can lead to unravel underlying structure of HA differentiation. Recently, a great attention has been paid to supervised machine learning methods implementing diverse amino acid composition and physic-chemical properties to unravel the underlying layers of protein function [21–31]. Mining of structural amino acid features have the potential to reflect these differences and lead us to specific changes which make a considerable impact on protein structure.

The determination of protein characteristics of HA subtypes in a comprehensive survey can provide a new vista for understanding the evolution of influenza based on the modulation of protein characteristics. Recent achievements in developing influenza forecasting mathematical models based on mining of massive number of emergency department visits [32] or monitoring the health-seeking behaviour of millions of users around the world in the form of queries to online search engines (particularly Google) [7] reinforce the necessity of large scale investigations in pattern recognition and modelling of influenza.

Machine learning methods have three main steps. The first step is extracting the n-dimensional features vector (which is composed of descriptors derived from the protein sequences in order to reflect different aspects of structural and physic-chemical properties of each protein) with a class label attached. Various sets of protein features including amino acid compositions, dipeptide compositions, pseudo amino acid compositions, normalized Moreau-Broto autocorrelation, Moran autocorrelation, Geary autocorrelation, and recently, distribution of various structural and physic-chemical properties have been used to make protein features vector [25,31,33–36]. Studies considering the impact of the different protein features in predicting protein function have demonstrated that the combination of protein features and considering features such as dipeptides gives a significantly higher performance than the use of individual protein features [36]. The second step of machine learning approach is application of machine learning method (or classifier) for prediction of the class label of the protein features input [25,26,31,33–36]. Currently, many machine learning methods, such as neural networks, support vector machine (SVM), and decision trees have been successfully developed for the prediction of protein function [21,22,25,27,30,37,38]. Each algorithm may be run with different criteria aiming to find important features and predict the function based on key announced features. The third step is measuring the performance of the prediction method and its validity using approaches such cross validation technique and independent evaluation (IE) datasets [39–50].

A decision tree is constructed by looking for regularities in data, determining the features to add at the next level of the tree using an entropy calculation, and then choosing the feature that minimizes the entropy impurity [51,52]. Decision tree is method of choice for prediction since it presents hierarchical ranking of important features and provides a clear image of differential protein structure [25].

Support Vector Machine (SVM) is a binary classification method proposed by Vapnik et.al (1995) which originally designed for classification and regression tasks [53]. The SVM method has been employed for pattern recognition problems in computational biology, including gene expression analysis [54], protein–protein interactions [55], protein fold class prediction [37,56], and protein–nucleotide interactions [21]. SVM has high performance level when high degree of diversity exists in datasets, because basically, SVM classifier depends on the support vectors, and the classifier function does not influenced by the entire dataset. Due to the exploitation of kernel functions, SVM is able to efficiently deal with a very large number of features [57]. Regarding the construction of a big dataset of protein features in this study, SVM is one of the preferred machine learning algorithms.

Bays and Empirical Bayes algorithms are highly efficient in prediction of cases with a Large number of variables but fewer observations [58]. Here, the dataset is unbalanced regarding the huge number of available sequences of H1 and H5 subtypes but small number of subtypes such as H15 and H16. In this case, *Naïve* Bayes based on Bayes conditional probability rule might work well in prediction.

Neural network is a mathematical structure able to process information through many connected neurons that respond to inputs through modifiable weights, thresholds, and mathematical transfer functions [59,60]. The network topology is one of the parameters that has a significant effect on the performance of a neural network [61]. Neural networks are widely used in a number of protein studies including protein secondary structure prediction [62], protein–nucleotide interactions [63], protein fold class prediction [37] and protein localization prediction [64].

Supervised machine learning algorithms have been merely applied on raw sequence. These modles have demonstrated high capability in rule discovery, finding hot spots on sequence, and prediction. Attaluri (2009) extracted 2154 sequences of H1, H2, H3, N1 and N2 antigens from NCBI and constructed J48 decision tree. They successfully identified 78 informative positions for detecting HA subtype and 63 positions for detecting NA subtype to predit virus subtypes [65]. In another study, protein sequences of all 8 segments of swine and human hosts of 2009 pandemics viruses were used to construct SVM model. The developed SVM model was able to detect viral host [66]. Association rules have been also employed in this context successfully to classify virus host between human, avian and swine [67].

Recently, we demonstrated that, instead of raw sequence analysis, extracting a large number of amino acid attributes and utilizing adequate data mining models can result in efficient and precise models in predicting the behaviour of malignant and benign breast cancer proteins [68], thermostable proteins [23], halostable proteins [69], ammonium transporters [70], and protein pumps [71]. A large scale analysis of amino acid structural

attributes of influenza surfaces proteins rather than raw sequence allignment, may provide a clearer image of underlying molecular mechanisms of host range increase by detecting the key structural protein characteristics which govern HA subtyping.

The aim of the present study was unravelling the molecular bases of HA subtype differences and discovery of the key protein characteristics which govern these differences. We used various clustering, screening, item set mining and decision tree models to determine which protein attributes may be used as a marker to differentiate between HA subtypes of influenza A viruses. A large scale analysis of computationally calculated protein characteristics of HA sequences provided a clear image of the role of simple amino acid characteristics in HA-based host differentiation. Finding reliable models to predict mutations/reassortments responsible for crossing the species barrier via amino acid features opens new avenues for prediction of protein structure of possible future pandemics.

Results

Data Cleaning

The initial dataset contained 7338 records (HA protein sequences) with 896 protein attributes. Of these records, 46.14% (3386 records) were classified as H1 class, 1.77% (130 records) as H2, and 26.06% (1913 records), 2.16% (159 records), 9.85% (723 records), 3.92% (288 records), 4.68% (344 records), 0.21% (16 records), 2.46% (181 records), 1.10% (81 records), 0.96% (71 records), 0.35% (26 records), 0.19% (14 records), 0.04% (3 records), 0.04% (3 records) were classified as H3 to H16, respectively. For each record, 868 features remained following removal of duplicates, useless attributes, and correlated features (dataset is available upon request).

When the number of variables (attributes) is sufficiently large, the ability to process units significantly reduces. Data cleansing algorithms have been used previously to remove correlated, superfluous or duplicated attributes and consequently to generate much smaller databases [27,28,72]. Following application of similar algorithms, 5% of the attributes were discarded from the original dataset.

Attribute Weighting

Data were normalised before running the models; consequently, all weights would be between 0 and 1. The result of application of 10 different attribute weighting algorithms is presented in Table S4. In this table, the weight closer to 1 shows high correspondence between the certain protein feature and target variable (HA subtypes). In other words, each weight shows the importance of each attribute regarding the target label based on its attribute weighting algorithm. As mentioned before, an attribute was assumed important if that attribute received weight higher than 0.5 (>0.5) by a certain attribute weighting algorithm (Table S4).

Weighting by PCA. Only one attribute, non-reduced Cys extinction coefficient at 280 nm significantly weighed (equal to 1.0, Table S4).

Weighting by SVM. The percentage of Met and Cys and the frequencies of Asp, Ala and Gln were the five protein attributes that gained weights higher than 0.50 (1.0, 0.89, 0.64, 0.54 and 0.54, respectively) (Table S4).

Weighting by relief. Thirteen attributes showed weights higher than 0.50 when this model was applied to the dataset (FCdb). These attributes were the frequencies of Phe – Ala and Tyr, the percentage of Tyr, the frequencies of Arg and Gln, the count of Ile, the percentage of Cys, the frequency of Glu, Isoelectric point, the frequency of Asp and Lys and the percentages of His and Try (Table S4).

Weighting by uncertainty. Sixteen protein attributes weighed higher than 0.50. The percentage and the frequency of Tyr gained the highest values (1.0 and 0.96, respectively) and the count of Thr gained the lowest value (0.51).

Weighting by gini index. Again the percentage and the frequency of Tyr weighed the highest (1.0) and the count of Ile, the frequencies of Arg and Gln, the count of Ser, the frequency of Glu, the percentage of Cys, the count of Val and nitrogen, the frequency of Asp, the percentage of His, weight, isoelectric point and the percentage of Try received the highest weighs as 0.95, 0.91, 0.87, 0.85, 0.81, 0.78, 0.77, 0.76, 0.72, 0.63, 0.52 and 0.51, respectively.

Weighting by chi squared. The following 13 attributes were weighted higher than 0.50: the percentage and the frequency of Tyr, the frequencies of Gln and Phe, non – reduced Cys extinction coefficient at 280 nm, the frequency of Gly, the percentages of Met and Cys, the frequency of Glu, the percentage of Try and Pro, the frequency of Ala and the percentage of His.

Weighting by deviation. We found the non – reduced Cys extinction coefficient at 280 nm to be the sole important protein attribute gained the weight equal to 1.0.

Weighting by rule. The frequencies of Tyr and Gln were among 14 other protein attributes with weighted equal to or greater than 0.50 when rule algorithm ran on the dataset. The other attributes were: the percentages of Tyr, Trp, Met, the frequencies of Phe and Arg, aliphatic index, the frequency of Glu, the count of Ile, the frequency of Asp, the count of Ser, isoelectric point and the frequency of negatively charged residues.

Weighting by Information gain. Seventeen protein attributes weighted equal to or greater than 0.50. They included the percentages of Cys and Tyr, the frequencies of Tyr and Glu, the count of Ile, the frequencies of Glu and Arg, the percentage of His, the counts of Ser, Nitrogen and Val, the frequency of Asp, weight, the percentages of Try and Meth, the count of hydrophilic residues, non – reduced Cys extinction coefficient at 280 nm and the frequency of Phe.

Weighting by Information gain ratio. When this algorithm was applied to the dataset, three attributes (the percentage of Try, the frequency of Ala and non – reduced Cys extinction coefficient at 280 nm) weighed the highest possible weights (1.0). Thirty other attributes had weights equal to or higher than 0.50.

Overall, the number of protein attributes that gained weights higher than 0.5 in each weighting model were as follows: *PCA* (1 attribute), *SVM* (5 attributes), *Relief* (13 attributes), *Uncertainty* (16 attributes), *Gini index* (15 attributes), Chi squared (13 attributes), Deviation (1 attribute), *Rule* (14 attributes), *Gain ratio* (33 attributes) and Info *Gain Ratio* (18 attributes). The most important attributes that were confirmed by different weighting algorithms to be involved in differentiation of HA protein are shown in Table 1.

Key Structural Protein Attributes Distinguishing Different Influenza HA Subtypes based on Overall Conclusion of the Attribute Weighting Algorithms

The protein attributes which announced important by most of the attribute weighting algorithms (intersection of different weighting methods) were assumed as the key distinguishing protein features in HA subtyping and are presented in Table 1. Based on the mentioned intersection of attribute weighting models (Table 1), 14 protein attributes were announced as the key distinguishing features in structure of HA subtypes, including The frequency of Gln, The frequency of Tyr, Percentage of Tyr, Percentage of Cys, The frequency of Glu, Percentage of Try,

Table 1. The most important protein attributes (features) in structure of different HA subtypes selected by different attribute weighting algorithms.

Attribute	The number of attribute weighting algoritms that indicated the attribute as important
The frequency of Gln	8
Percentage of Cys	7
Percentage of Tyr	7
The frequency of Tyr	7
The frequency of Glu	7
Percentage of Try	7
Count of Ile	6
The frequency of Arg	6
Percentage of His	6
The frequency of Asp	6
Percentage of Met	6
Non-reduced Cys extinction coefficient at 280 nm	6
The frequency of Phe	5

Total number of attribute weighting algorithms which have announced the certain attribute important (weight higher than 0.5, Table S4). This table presents the number of algorithms that selected the attribute. Weighting algorithms were PCA, *SVM*, *Relief*, *Uncertainty*, *Gini index*, *Chi Squared*, Deviation, *Rule*, Information Gain, and Information Gain Ratio.
doi:10.1371/journal.pone.0096984.t001

Count of Ile, The frequency of Arg, Percentage of His, The frequency of Asp, Percentage of Met, Non-reduced Cys extinction coefficient at 280 nm and The frequency of Phe.

Decision Trees

Of the 176 generated trees, most of them generated good and meaningful trees and just one of them (*Random Tree with accuracy criteria*) did not result in tree with roots and leaves.

The count of Tyr was the sole attribute used to build a single - branched tree when the *Decision Stump* model on *Gini Index* criterion was applied to the dataset (Figure 1). When the value for this feature was equal to 26, 27, 28, 29 or 30, the virus fell into the H1 class; if the value was equal to 18, 19, 20, 21 or 22, the virus belonged to the H3 class. When the count of Tyr was equal to 17, the subtype of the virus was H4; but when the value was equal to 23 or 24, the virus was associated with H5. H6 virus subtype was identified when the count of Tyr was equal to 26. Finally, when the value was equal to 13, 14 or 15, the virus fell into the H7 class.

Other simple trees were produced by the *Decision Stump* and *Decision Tree* models on the accuracy criterion with just one branch. The frequency of positively charged residues was the sole protein attribute used to build this tree. Similarly the *Decision Tree Parallel* (run on *Gain Ratio* criterion) generated a tree with one branch. This branch is based on the frequency of Pro – Ala with a frequency equal to or less than 0.5 indicating a H1 subtype. The *Decision Stump* (run on gain ratio) model induced a one-level tree showing the percentage of Try to be the most important feature. Specifically, when the value for this feature was higher than 1.407, the virus fell into the H1 subclass, but when the value was equal to or higher than 1.407, the virus belongs to the H5 class. Higher power of trees in distinguishing H1 and H5 is because of higher numbers of these subtypes and consequently, ans consequently better training and higher capability of theses models in recognition of H1 and H5.

In other decision trees such as those produced by the *Random Tree* (on *Gini index*), dipeptide features (such as the frequency of Pro – Gly) were the main protein feature to build the trees and the following protein attributes were used to build the tree branches: the count of Phe – Met, the count of Asn – Met and the frequency of Trp - Leu. All virus subclasses (except H6, H8, H10, H11 and H14) were classified by this model (Figure 2). This model was one of the most successful models in distinguishing H subtypes.

Decision Tree Parallel model (run on *Information gain*, *Gini index* or *Accuracy* criteria), *Decision Tree* model (run on *Information gain*, *Gini index* or *Gain ratio*) and *Random Tree* model (run on *Information gain*) induced very complex trees to distinguish between all virus subclasses using protein attributes.

Three models of *Decision Stump* (*Gain ratio*, *Gini Index* and *Accuracy*) induced trees with just one branch, and the percentage of Try, the percentage of Tyr and the frequency of Glu were the most important protein attributes, respectively. *Decision Tree* model (run on *Accuracy*) built a two branches tree; if the frequency of Glu was equal to or less than 0.054, the virus fell into H3, but when the value for this feature was higher than 0.054 and the weight of protein was higher than 63.971, the virus belonged to H5. If the weight was equal to or less than 63.971 and the frequency of Tyr was higher than 0.028, the virus was from the H1 class; otherwise from the H7 class. The other models induced more complex tress.

The percentage of Tyr, the percentage of Try and the frequency of Glu were the most important features selected by *Decision Stump* models to induce a simple tree with one branch. A two branches tree induced by *Decision Tree* model (run on *Accuracy*), and the frequency of Glu and the weight and non – reduced Cys extinction coefficient at 280 nm were the key protein attributes to classify H1, H3, H5 and H7. The other models induced more complex tress. Again, *Stump Decision* models induced simple trees and the count of other residues, the percentage of Tyr and the percentage of Try were the most important features to build the trees on the *Info Gain* Ratio database. The accuracies of different decision tree models are compared in Table 2.

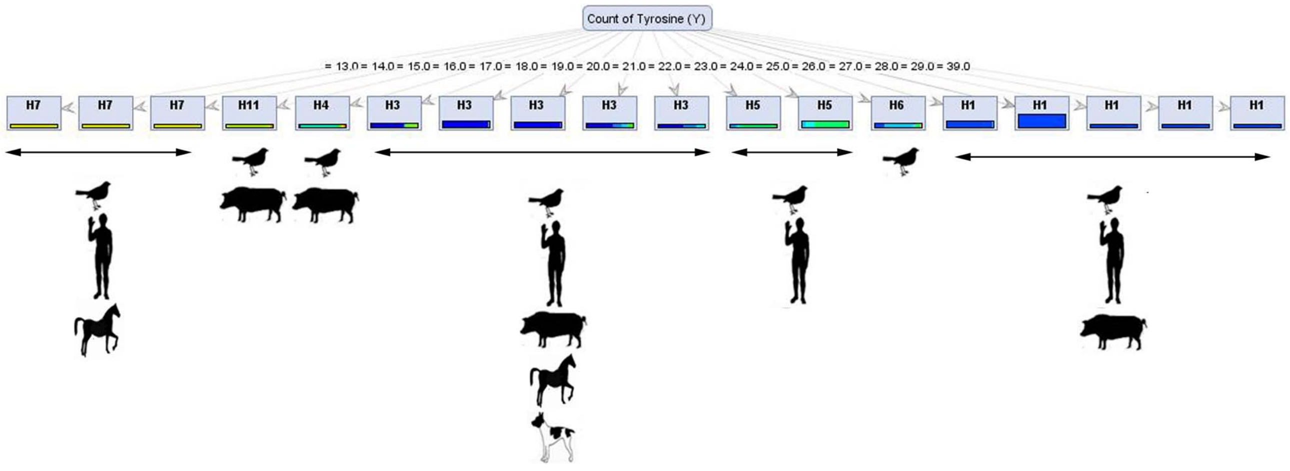

Figure 1. *Decision Tree* from *Decision Stump* model ran with *Gini Index* criterion. As may be inferred from the figure, the count of Tyr was the most important and the sole protein attributes in distinguishing various HA subtypes of influenza virus A. When the value for this feature was equal to 26, 27, 28, 29 or 30, the virus fell into the H1 class; if the value was equal to 18, 19, 20, 21 or 22, the virus belonged to the H3 class. While the count of Tyr was equal to 17, the subtype of the virus was H4; but when the value was equal to 23 or 24, the virus was associated with H5. H6 virus subtype was identified when the count of Tyr was equal to 26. Finally, when the value was equal to 13, 14 or 15, the virus fell into the H7 class. Underneath, the host species for each virus class has been depicted.
doi:10.1371/journal.pone.0096984.g001

Machine Learning Models to Predict Unknown Influenza A Virus Classes Based on Protein Attributes

Decision trees. The accuracies of trees induced by various decision tree models are presented in Table 2. Generally, most models showed accuracies higher than 80% while the lowest accuracies gained by *Decision Tree* models ran on *FCdb* (the original) dataset with *Info Gain* criteria (average of 46.04%). The best predicted accuracy achieved when *Decision Tree* run with *Gini Index* criteria on *Uncertainty* dataset (99.70%) (Figure 2).

SVM approach. The total accuracy predicted by *C-SVC* method (when *Gamma* and *C* were 0.0065 and 10, respectively) reached 94.52% ±0.86% with the *RBF kernel* function (the lowest and highest prediction rates − 65.19% and 100% − obtained for

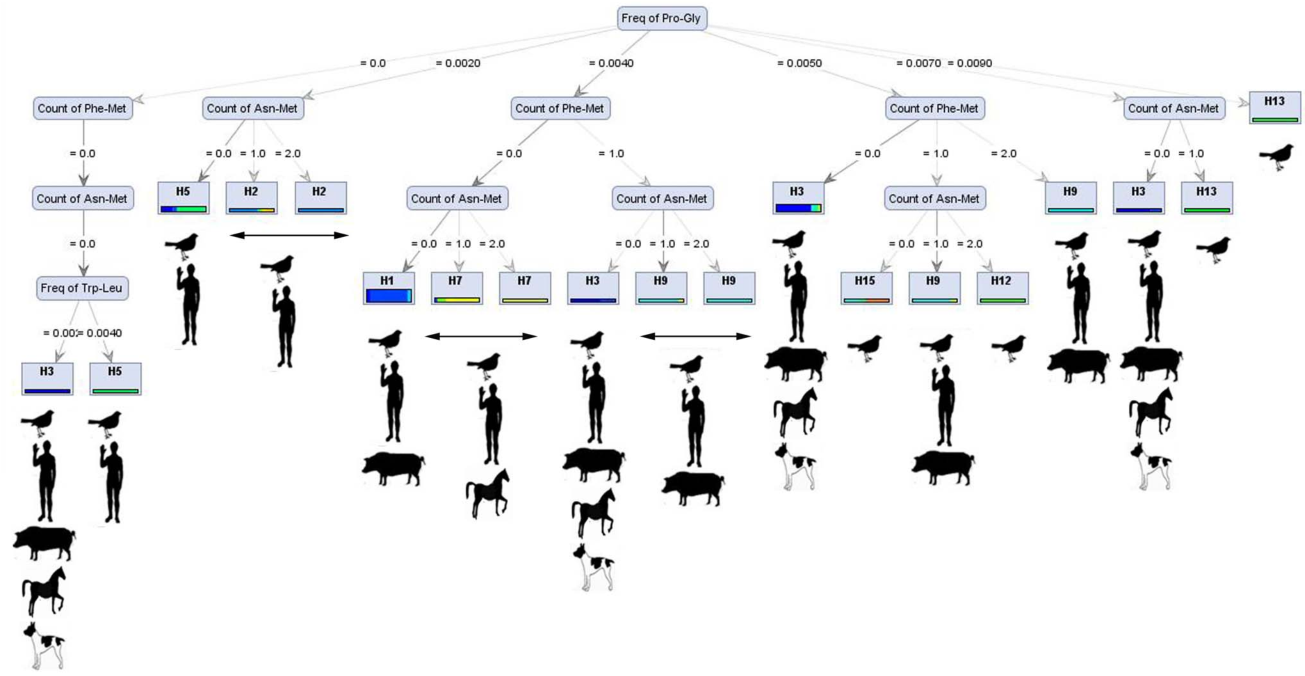

Figure 2. *Decision Tree* from *Random Tree* model ran with *Gini Index* criterion. As may be inferred from the figure, the frequency of Pro - Gly was the most important protein attributes to build the tree and the counts or the frequencies of other dipeptides used to generate the tree branches and to distinguish various HA subtypes of influenza virus A. With the defined value for the count of Phe – Met, the count of Asn – Met and the frequency of Trp – Leu, the virus subtypes were either H3 or H5. With different values for the count of Asn – Met, various virus subtypes distinguished. All virus subclasses (except H6, H8, H10, H11 and H14) were classified by this model. Underneath each subtype common host has been depicted.
doi:10.1371/journal.pone.0096984.g002

Table 2. The accuracy of four different tree induction models (each ran with four criteria, *Accuracy, Gain Ratio, Gini Index and Info Gain*) on 11 datasets [original protein features dataset (FCdb) as well as 10 datasets generated by trimming (filtering) the original FCdb dataset by attribute weighting algorithms) computed by 10-fold cross validation.

	Decision Tree				Decision Tree Parallel				Decision Tree Stump				Decision Tree Random Forest			
Dataset Filtered by	*Accuracy*	*Gain Ratio*	*Gini Index*	*Info Gain*	*Accuracy*	*Gain Ratio*	*Gini Index*	*Info Gain*	*Accuracy*	*Gain Ratio*	*Gini Index*	*Info Gain*	*Accuracy*	*Gain Ratio*	*Gini Index*	*Info Gain*
Chi Squared	99.52	99.52	99.52	99.52	94.22	99.27	99.46	99.47	71.63	48.48	71.18	71.98	88.90	91.72	99.51	99.54
Info Gain	99.33	99.33	99.33	99.33	89.30	99.56	99.51	99.33	71.63	49.80	71.18	71.98	86.18	95.92	99.52	99.44
**Deviation**	70.73	70.73	70.73	70.73	55.01	70.73	96.78	96.80	50.65	50.73	58.85	58.85	55.13	70.96	96.65	96.80
Gini Index	99.24	99.24	99.24	99.24	89.19	99.39	99.29	99.32	71.63	49.80	71.18	71.98	82.47	94.45	99.43	99.14
***Info Gain* Ratio**	99.43	99.43	99.43	99.43	91.69	99.40	99.35	99.25	71.63	48.48	71.18	71.98	87.37	93.12	99.01	99.33
**PCA**	70.73	70.73	70.73	70.73	55.01	70.73	96.78	96.80	50.65	50.73	58.85	58.85	55.13	70.96	96.65	96.80
Relief	99.35	99.35	99.35	99.35	87.58	99.31	99.37	99.22	71.63	49.80	71.18	71.98	87.88	96.64	99.24	98.87
Rule	99.47	99.47	99.47	99.47	91.36	99.37	99.24	99.39	71.63	49.80	71.18	71.86	82.51	93.81	99.36	98.86
Uncertainty	99.40	99.40	99.40	99.40	91.70	99.40	99.32	99.25	71.63	49.80	71.18	71.98	88.62	95.01	99.70	99.65
SVM	98.99	98.99	98.99	98.99	93.64	92.48	99.09	99.09	70.99	46.40	70.05	72.03	86.13	89.00	98.56	98.06
FCdb (original protein features dataset)	99.06	96.87	74.36	46.04	76.13	96.87	74.93	74.50	48.48	90.00	58.38	58.38	97.65	99.31	98.34	97.73

This table presents the accuracy percentage of Tree Induction models (*Decision Tree, Decision Tree Parallel, Decision Stump, Random Forest and Random Tree*) run with four different criteria (*Gain Ratio, Information Gain, Gini Index and Accuracy*). The lowest and highest accuracies have been highlighted.

doi:10.1371/journal.pone.0096984.t002

H5 and H1, respectively). The average number of support vectors was 161.88 with the lowest number observed for class H15 and H16 (3) while the highest number of support vectors was gained for H3 (527).

When *Gamma* and *C* changed to 0 and 10, the total prediction accuracy reached to 99.70% ±0.15%. The average number of support vectors for this run decreased to 54.50 with the lowest number (3) again for the same classes (H15 and H16) and the highest number for H1 class (181). The accuracy did not improve by using the other kernel functions (linear, poly, sigmoid, and pre-computed).

Naïve bayes. As seen in Table 3, the lowest accuracy (63.36%) gained when *Naïve Bayes* model ran on *SVM* dataset. In contrast, the best accuracy (99.22%) gained when Bayes Kernel model ran on *FCdb*. The accuracy of *Naïve Bayes* model ran on *FCdb* was 98.95% ±0.30% while the same accuracy for *Naïve Bayes* Kernel was higher (99.27% ±0.20%). Distribution model for label attribute (virus subtype) ranged from 0.0001 for H15 and H16 classes up to 0.460 for H1 classes for both *Naïve Bayes* and *Naïve Bayes* Kernel.

Neural network. Tables 3 presents the comparative performances of different combinations of neural network algorithms with 11 datasets [original dataset (FCdb) as well as datasets pre-filtered by PCA, Uncertainty, Relief, Chi Squared, Gini Index, Deviation, Rule, Gain Ratio, Info Gain, and SVM weighting algorithms based on 10-fold cross validation. The average accuracies of *Auto MLP* and *Neural Net* models were generally high (more than 95%, presented in Table 3).

As it can be inferred from Table 3, pre-trimming of protein features dataset with proper attribute weighting algorithm plays a determining role in obtaining high prediction accuracy. When the models ran on *PCA* databases, the accuracies were minimum (78.81% and 81.44%, respectively), while the best accuracy (99.73%) gained when either *Neural Net* or *Auto MLP* models ran on *Info Gain* Ratio dataset or *Chi Squared* datasets. The performances of neural network models on other datasets (such as *Gini Index*, *Info Gain*, *Relief*, *Rule*, *SVM*, and *Uncertainty*) were also higher than 99% showing their suitability on predicting the right HA virus classes.

Statistical Analysis of the Key Found Protein Attributes in the Structure of HA Subtypes

Statistical distribution of important key amino acid attributes in the structure of HA subtypes is presented in Table S2. Interestingly, The freq of Glutamine and The freq of Tyrosine which were selected by most of attribute weighting algorithms (Table 1) and decision tree models (Figure 1) had the highest variation between HA subtypes. This confirms the high efficiency of attribute weighting algorithms in feature selection and finding the effective features.

In agreement with the outcomes of attribute weighting algorithms, ANOVA showed that that in respect to different HA subtypes (as dependent variable), differences between important structural features including Non-reduced cysteines Extinction, Count of Isoleucine, Freq of Cysteine, Freq of Aspartic Acid, Freq of Glutamic Acid, Freq of Glutamine, Freq of Arginine, Freq of Tyrosine, Percentage of Histidine, Percentage of Methionine, Percentage of Tryptophan, Percentage of Tyrosine, Count of Phe-Met, Freq of Pro-Gly, and Freq of Trp-Leu are highly significant ($p = 0.01$) (Table S3). Interestingly, the Freq of Glutamine and Freq of Tyrosine received the highest R-square (95.75 and 96.47, respectively) implying that these two attributes, highlighted by decision tree and weighting algorithms (Figure 1 and Table 1), are the key predictors of HA subtyping. It should be mentioned that higher value of R-square denotes that higher amount of variance of dependent variable (HA subtypes) is related to independent variable (Freq of Glutamine and Freq of Tyrosine).

MANOVA (multivariariate analysis) also confirmed the overall significant differences ($p = 0.05$) of the selected features between different HA subtypes (Table S3). Clustering, based on the 16 important features is presented in Figure S1. As it can be inferred in Figure S1, the key discovered features in this study have highly accuracy in prediction and separation of HA subtypes and reinforces the high capability of discovered features as predictors of HA subtyping and underlying layer of subtype differentiation.

Table 3. The accuracy of Baysian and Neural Network models on various datasets [11 datasets including original protein features dataset (FCdb) as well as 10 datasets generated by trimming (filtering) the original FCdb dataset by attribute weighting algorithms] computed by 10-fold cross validation.

	**Baysian Models**		**Neural Nets Models**	
	Bayse Kernel	*Naive Bayse*	*Auto MLp*	*Neural Net*
Chi Squared	98.72%	98.59%	99.71%	99.73%
**Deviation**	88.55%	96.70%	79.94%	82.71%
Gini Index	98.90%	98.26%	99.70%	99.69%
Info Gain	98.12%	98.79%	99.70%	99.70%
***Info Gain* Ratio**	84.38%	63.36%	99.70%	99.73%
**PCA**	99.22%	98.69%	78.81%	81.44%
Relief	98.95%	98.65%	99.63%	99.59%
Rule	98.53%	98.97%	99.70%	99.67%
SVM	84.38%	63.36%	98.44%	98.37%
Uncertainty	92.69%	97.89%	99.71%	99.71%
FCdb	%99.18	%97.51	%99.73	99.69%

This table presents the accuracy percentage of Baysian (*Naïve Bayes and Bayse Kernel*) and Neural Network models (*Auto MLp and Neural Net*) run on all 10 datasets.
doi:10.1371/journal.pone.0096984.t003

Discussion

In the last century, three influenza A pandemics happened: H1N1 in 1918, H2N2 in 1957 and H3N2 in 1968 [73]. Since 1997, avian H5N1 and recently avian H7N9 influenza have been infecting humans zoonotically with high mortality rates. The 2009 pandemic was the result of H1N1 multiple reassortant with genes derived from viruses that originally circulated in the swine, avian, and human populations [74]. 2013 China pandemic was the result of novel reassortant in avian influenza (H7N9) with six internal genes from avian influenza A (H9N2) viruses. Substitution Q226L (H3 numbering) at the 210-loop in the HA gene was found in two pandemic strains [3]. To be prepared for future pandemics, a detailed understanding of the basic biology of this virus its evolution and subtype differentiation is critical.

An important point of this study was discoverey of some new rules underpining HA differentiation, not in the level of sequence but in the level of structural protein architechture via extraction and data mining of structural protein features. Influenza virus is also subjected to host immune pressure (enriched by vaccines in many cases) and undergoes rapid evolution in the antigenic regions, especially when the virus crosses the host species barrier [75]. Influenza virus does not have enough time to develop/add long domains or major changes in proteins; instead, it is reasonable that influenza viruses alter short amino patterns. Consequently, rapid structural amino acid alteration is an strategy for influenza to survive. Based on the key amino acid attributes, we found the possible routes (based on decision tree algorithms) that influenza can acquire additional host specificity of another subtype via a small change in amino acid attributes (Figure 2). Through theses routes, influenza has more chance in increasing host range and survival by acquiring host specificity of another subtype with less energy consumption and minimum change in protein structure.

This is the first successful high accurate attempt in modelling and prediction of influenza A virus subtypes based on physic-chemical properties of HA proteins. It should be noted that unbalanced number of HA subtypes can influence the efficiency of attribute weighting and modelling. Considering that each attribute weighting system uses a specific pattern to define the most important features, results may vary according to modelling techniques as has been highlighted in previous studies [23,27,28,72,76,77]. Despite this possibility, our analysis revealed a high level of accuracy. Applying 10 statistically different attribute weighting algorithms and selection of the key features based on the overall (intersection) of these algorithms reinforce the importance of the selected features. Furthermore, weighting algorithms such as Relief can deal with unbalanced data by taking subsamples and reduce this bias [78]. Altogether, achieving the precise modelling approach in linking the amino acid attributes with HA subtyping is the result of the following improvements in the present study: (1) increasing the number of computationally calculated amino acid features to cover different aspects of HA protein structure, (2) testing different feature selection (attribute weighting) algorithms and selection of the most important amino acid attribute based on the overall conclusion of algorithms, (3) examining different supervised data mining (machine learning) algorithms, and (4) joining attribute weighting with different data mining algorithms which sharply increases the accuracy of the models in some cases.

The frequency of Gln was the most important feature to distinguish virus subtypes, as defined by 80% of the attribute weighting algorithms while the percentages of Cys, Tyr and Try and the frequencies of Tyr and Glu were defined as important features by 70% of algorithms (Table 1). Gln, Cys, Tyr and Glu have been classified as polar or hydrophilic amino acids. Thus, our results confirm the importance of hydrophilicity in forming HA proteins. In fact, hydrophobicity has been used as a vital parameter in designing a new anti-viral vaccine against HA proteins [79]. It has been shown that an increase in the hydrophilicity of the receptor binding region is apparently an evolutionary adaptation of the 2009 H1N1 pandemic influenza A virus in 1918 Spanish, 1930 swine, and 2005 seasonal strains [80]. It has also been identified that mature peptide sequences of HA genes isolated from humans in 2009 have Gln at position 226 of the receptor binding site [81]. Interestingly, this was different from previously isolated viruses where the presence of Leu at the same position contributes to a preference for human receptors whereas the presence of Gln contributes to a preference for avian receptors [81].

Some generated models, such as *Decision Tree* from *Random Tree* model ran with *Gini Index* criterion (Figure 2), provide novel knowledge in structural amino acid architecture and packaging of different HA proteins. It shows that count of specific dipeptides including Phe-Met and Asn-Met structurally differentiate H1 from H3. Theses dipeptides are the possible modulated amino acid features in the structure of future Influenza A viruses tending to acquire host range of both H1 and H3 subtypes. Figure 2 (left branch of tree) predicts that mutation or reassortment in H5 resulting in lower number of Trp-Leu which may be considered as a key requirement for increasing host range and possible future pandemics by H5 subtype. Similar conclusion has been made recently documenting that the A/Indonesia/5/2005 avian A/H5N1 influenza virus may require as few as five amino acid substitutions [17,82] and the A/Vietnam/1203/2004 requires four substitutions and reassortment [14,18] to become transmissible between ferrets via respiratory droplets. Interestingly, Russell et al., 2012 demonstrated that these substitutions can arise in nature [19]. Based on the above discussion, the presented bioinformatics models in this study may open a new avenue in predicting the structure of future influenza A pandemics.

Up to now, there has been little discussion about the role of di-peptides in protein function. Our recent study has already demonstrated that specific di-peptides play the central role in protein halostability and thermostability [28,72]. The role of some di-peptides, such as Pro - Ala, Pro – Gly, Phe – Met, Trp – Leu and Trp – Pro (selected as important protein features by decision tree models to identify influenza A subtypes) was indentified in the present study. Interestingly, as presented in Figure 2, wide-speared HA subtypes of influenza virus (H3 and H1) have lower frequency/counts of particular dipeptides such as Asn-Met, Phe-Met, and Pro-Gly. Decreasing the number of tough structural dipeptides provides more flexibility for virus to attack surface cells of different hosts and escap host immune system.

The ability of various decision tree induction models applied in this study to correctly classify influenza A subtypes based on protein attributes varied considerably. In some models, very few subtypes (two or three) were identified which illustrate the incompetence of these models. However, other models, such as *Stump* and *Random tree* run on removed correlated features dataset were able to completely classify the HA subtypes based on their protein features. The latter models are suitable tools to classify those viral subtypes. As shown in Table 2, the overall accuracies for tree induction models were generally high and improved when joined with appropriate feature weighting criteria. For example, the accuracy for *Decision Tree* model with *Info Gain* criterion run on FCdb was 46.04%, but improved to 99.06% when the criterion changed to *Accuracy*, indicating a very sharp increase in the model accuracy and performance. The best accuracy achieved when the

Random Forest model ran with *Gini Index* criterion (99.70%) which makes it the best model to apply in such conditions. The performances of *Naïve Bayes* models (with and without kernel) were also high enough (more than 99%), which also makes these models to cluster HA subtypes into the right classes with high accuracies.

SVM is a supervised non-parametric statistical learning technique with there are no assumption problem that usually involve in identification of multiple classes (more than two). Adjustments are made to the simple *SVM binary* classifier to operate as a multi-class classifier using methods such as one-against-all, one-against-others, and directed acyclic graph. Since example data is often not linearly separable, *SVM*s introduce the notion of a "*kernel induced feature space*" which casts the data into a higher dimensional space where the data is separable. Overall, *SVM*s are intuitive, theoretically well- founded, and have been shown to be practically applicable. The methods have been widely employed by researchers in different areas of science [83–88], including influenza research [89–93]. In general, the *RBF kernel* is a reasonable first choice in *SVM* approaches. This kernel nonlinearly maps samples into a higher dimensional space. Thus, unlike the linear kernel, it can handle the situation when the relationship between class labels and attributes is nonlinear [93]. In this study, *RBF kernel* function applied and showed high level of accuracy in classifying 16 classes of HA viruses based on their protein features. These results illustrate for the first time that *RBF kernel* is an appropriate model (on dataset scaled by grid search) to find the key underlying physic-chemical charateristics of HA subtypes and predict based on them. Convergence of training with SVM (which is a deterministic quadratic optimization procedure) is much faster than neural network (which is randomized procedure).

Sequence-alignment based methods (BLAST and phylogenic trees), drawn by nucleic acid or amino acid sequence alignments, have been extensively employed as the basis for evolutionary studies. However, homology-based methods does not consider the structural and functional features of proteins during evolution [24]. The presented approch in this study based on the machine learning algorithms running on structural protein features provides a new evolutionary pathway separation of HA a subtype which takes into account the structural reasons of this diversity. As discussed before [24], the presented procedure can significantly enrich and qualify any type of further evolutionary studies by completion of the common sequence homology based phylogenic analysis methods.

There is a major difference between the presented method in this study with the "composition vectors based methods" as another alignment-free method. The "composition vectors based methods" use a sting or repeats (amino acid or nucleotide), with a limited number between 1 to in maximum 7 and count the number of these repeats within different genomes or genes [94–96]. If we consider each repeat as a feature (attribute); in total, we will have 7 features. In our method, "large scale supervised mining of protein attributes", we have calculated 896 physic-chemical protein characteristics for each sequence which offers a comprehensive view on underlying protein architecture and shed light on the key protein characteristics which govern HA subtyping.

This research will serve as a basis for future studies on prediction of the structure of future HA sequences which will be capable of infecting a broad range of hosts. Applying this approach on other influenza protein segments in particular N and M2e (the other surface protein) will complete the puzzle of underling structural protein architecture of influenza subtypes and the possible structural changes which happens during host range increase.

Based on the findings of this study, it is possible to predict antigenic variation of any input influenza sequence using haemagglutinin amino acid composition. This prediction does not need similarity searches or gathering information about the complex, expensive, and time-consuming features of the tertiary and quaternary protein structure or any need to laboratory activity. The developed models can be further embedded in web-based applications or softwares to predict possible pandemic strains from recently observed or computationally guessed HA sequences. Extraction of a large number of protein characteristics and pattern recognition trough supervised machine learning models can also be employed in future studies on understanding the interaction between antigen and antibody (immunity reactivity measured by ELISA/Western Blot [97]) and finding the key underlying physic-chemical characteristics in the structure antibody and antigen which can result in vaccine breakdown.

In addition, our findings add to the growing body of literature on the molecular biology of influenza virus, which are urgently needed by many industries and vaccine-producing institutes. Furthermore, our method will serve as a model for future investigation on NA and M2e amino acid compositions, as well as the interaction between HA, M2e, and NA antigens. This study suggests that amino acid profiling of influenza surface proteins has the potential to monitor host specificity.

Materials and Methods

i: Extraction of Structural Protein Attributes Based on HA Sequences

Seven thousand and three hundred and thirty eight sequences of HA virus proteins from various species (human, bird, pig, horse, mouse, etc) were extracted from the Influenza Research Database (http://www.fludb.org/) and categorized as H1 to H16, according to the database classification. Some sequences of avian influenza were obtained based on AH/2006/050 and AH/2010/039 projects supported by Australian Centre for International Agricultural Research (ACIAR).

For each of these sequences, eight hundred and ninety six protein features (attributes) such as length, weight, isoelectric point, count and frequency of each element (carbon, nitrogen, sulphur, oxygen, and hydrogen), count and frequency of each amino acid, count and frequency of negatively charged, positively charged, hydrophilic and hydrophobic residues, count and frequency of dipeptides, number of α-helix and β-strands, and other secondary protein features were extracted using various bioinformatics tools and software from the ExPASy site (http://www.expasy.org) and CLC bio software (CLC bio, Finlandsgade 10–12, Katrinebjerg 8200 Aarhus N Denmark). All features were classified as continuous variables, except virus subtypes, virus host and N-terminal amino acids, which classified as categorical. A dataset of these protein features imported into RapidMiner software [RapidMiner 5.0.001, Rapid-I GmbH, Stochumer Str. 475, 44227 Dortmund, Germany]. Virus subtypes (H1-H16) was set as the output variable (label) and the other variables were set as input variables. The following steps were then applied to the dataset.

ii: Data Cleaning

Initially, duplicate features were removed by comparing all examples on the basis of the specified selection of attributes (two examples were assumed equal if all values of all selected attributes were equal). Then, we removed the superfluous attributes from the dataset. Nominal attributes were regarded as superfluous when the most frequent values were contained in more or less than nominal

useless above or below percent of all examples. Numerical attributes which possessed standard deviations less than or equal to a given *deviation* threshold (0.1) were assumed to be superfluous and removed. Finally, correlated features (with Pearson correlation greater than 0.9) were omitted. After cleaning, the number of attributes decreased from 896 to 893. We called this dataset final cleaned dataset (*FCdb*) and used for next attribute weighting analysis. This dataset is represented in Table S1.

iii: Application of Attribute Weighting Algorithms to Find the Most Important Structural Protein Attributes Distinguishing HA Subtypes

To determine how structural protein features of HA sequence determines its final subtype, 10 different attribute weighting algorithms including weighting by PCA, weighting by Uncertainty, weighting by Relief, weighting by Chi Squared, weighting by Gini Index, weighting by Deviation, weighting by Rule, weighting by Gain Ratio, weighting by Info Gain, and weighting by SVM were applied on final cleaned database (*FCdb*, Table S1). Attribute weighting algorithms find the most important protein attributes which differ in protein structure between different HA subtypes. The protein attributes which announced important by most of attribute weighting algorithms (intersection of different weighting methods) were assumed as the key distinguishing features of HA subtypes.

Ten new generated datasets produced by trimming (filtering) of the original dataset (*FCdb*) via attribute weighting algorithms as well as the original FCdb dataset (11 datasets in total) were used as input for Decision Tree, Baysian, and Neural Network models.

Weight by Information gain. This operator calculated the relevance of a feature by computing the *Information Gain* in class distribution.

Weight by Information Gain ratio. This operator calculated the relevance of a feature by computing the information *Gain Ratio* for the class distribution.

Weight by rule. This operator calculated the relevance of a feature by computing the error rate of a one R Model on the example set without this feature.

Weight deviation. This operator created weights from the standard deviations of all attributes. The values were normalised by the average, the minimum, or the maximum of the attribute.

Weight by chi squared statistic. This operator calculated the relevance of a feature by computing, for each attribute of the input example set using chi-squared statistic with respect to the class attribute.

Weight by gini index. This operator calculated the relevance of an attribute by computing the *Gini index* of the class distribution, if the given example set would have been split according to the feature.

Weight by uncertainty. This operator calculated the relevance of an attribute by measuring the symmetrical uncertainty with respect to the class.

Weight by relief. This operator measured the relevance of features by sampling examples and comparing the value of the current feature for the nearest example of the same and of different class. This version also worked for multiple classes and regression data sets. The resulting weights were normalised into the interval between 0 and 1.

Weight by SVM (Support Vector Machine). This operator used the coefficients of the normal vector of a linear *SVM* as feature weights.

Weight by PCA (Principle Component Analysis). This operator used the factors of the first of the principal components as feature weights.

iv: Attribute Selection and Generation of New Pre-trimmed Cprotein Feature Datasets

After running attribute weighting models were run on FCdb dataset (original clean dataset of protein features), each protein attribute (feature) gained a value between 0 and 1, which reveales the importance of that attribute with regard to a target attribute (HA subtypes). As mentioned before, an attribute was assumed important if that attribute received weight higher than 0.5 (>0.5) by a certain attribute weighting algorithm (Table S4). The protein attributes which announced important by most of attribute weighting algorithms (intersection of different weighting methods) were assumed as the key distinguishing protein features in HA subtyping and are presented in Table 1. In conclution.

All variables with weights higher than 0.50 were selected and 10 new datasets were created according to 10 applied attribute weighting algorithms. These newly formed datasets were named according to their attribute weighting models (*Information gain, Information gain ratio, Rule, Deviation, Chi Squared, Gini index, Uncertainty, Relief, SVM and PCA*) and were used to join with subsequent models (supervised). Each model of supervised or unsupervised clustering were performed 11 times; the first time it ran on the main dataset (*FCdb*) and then on the 10 newly formed datasets from attribute weighting and selection.

V: Machine Learning Models to Predict Unknown Influenza A Virus Classes Based on Protein Attributes

As mentioned above, the original FCdb dataset as well as 10 new generated datasets produced by trimming (filtering) of the original dataset (*FCdb*) via attribute weighting algorithms were used as input for machine learning models.

Four classes of machine learning models (Decision Trees, SVM, Baysian and Neural Network algorithms) were applied on all 11 datasets to find suitable model(s) in order to predict unknown classes of influenza A virus virus based on the computed protein attributes computed.

To prevent overfitting and calculate the accuracy of each model, 10-fold cross validation was employed in this study to train and test models on all patterns. Ten-fold cross validation is a standard and commonly used method for evaluating classifier methods, as set of proteins used for training and testing are mutually exclusive. Details of this well-known method and its principals have been extensively discussed in many previous published papers [18–29].

Based on 10-fold cross validation; records were divided into 10 nearly equal parts randomly. Our dataset had 7353 records in total. In other words, records (7353) were randomly divided into 10 parts: 9 parts consisted of 735 records, and the last one contained 738 records. When prediction algorithms such as SVM, Neural Network or Tree Induction models were performing, nine sets (parts) were used for training and the 10th one for testing. Then, in next run, another part set as testing set and the other 9 parts as training sets. The process was repeated 10 times and the accuracies for true, false and total accuracy were calculated. The final accuracy reported as the average of the accuracy in all ten tests (runs).

In each run, the predictor or machine learning system did not expose to test set and just trains on 9 training sets (so this diminish the possibility of overfitting into zero). When the system is trained well and the calculated accuracy is reached at least to 0.85%, then the model tries to predict or guess the unknown test set and calculates the accuracy for this set. Repeating the same procedure for 10 times and non-using of the test set for training are the bases of preventing the learning application from overfitting.

Decision tree. Sixteen machine learning models run on four decision tree algorithms (*Decision Tree, Decision Tree Parallel, Decision Stump and Random Forest*) with four different criteria (*Gain Ratio, Information Gain, Gini Index and Accuracy*) on all 11 datasets. A decision stump is a Decision Tree, which uses only a single attribute for splitting. For discrete attributes, this typically means that the tree consists only of a single interior node (i.e., the root has only leaves as successor nodes) [98]. If the attribute is numerical, the tree may be more complex. Models trained and tested with ten-fold cross validation and the average of accuracies were computed as stated above.

Support Vector Machine (SVM). *SVMs* are popular and powerful techniques for supervised data classification and prediction, so four different *SVM* models (*SVM, LibSVM, Linear SVM and Evolutionary SVM*) were used here to implement models for prediction of Influenza A classes based on protein features. Briefly, all 11 databases [original protein feature dataset (FCdb) and 10 datasets generated by trimming (filtering) FCdb dataset by PCA, Uncertainty, Relief, Chi Squared, Gini Index, Deviation, Rule, Gain Ratio, Info Gain, and SVM weighting algorithms] were transformed to *SVM* format and scaled by grid search (to avoid attributes in greater numeric ranges dominating those in smaller numeric ranges) and to find the optimal values for operator parameters.

To prevent overfitting problems, again 10-fold cross validation was applied and the averages of accuracies were computed. *RBF kernel* which nonlinearly maps samples into a higher dimensional space and can handle the case when the relation between class labels and attributes is nonlinear used to run with *SVM* models. Grid search is an appropriate way to determine the optimal values for two major parameters of the RBF (parameters C and gamma). Other kernels such as linear, poly, sigmoid and pre-computed were also applied to the datasets to find the best accuracy.

Naïve bayes. *Naïve* Bayes based on Bayes conditional probability rule was used for performing classification and prediction tasks. *Naïve Bayes* assumes the predictors are statistically independent which makes it an effective classification tool and easy to interpret. Two models, *Naïve* base (returns classification model using estimated normal distributions) and *Naïve* base kernel (returns classification model using estimated kernel densities) trained with 10-fold cross validation on all 11 databases (original dataset as well as 10 dataset generated by applying 10 different attribute weighting algorithms) and the model accuracies in predicting the right HA virus class computed as stated before.

Neural network. Two neural networks models (*Neural Net and AutoMLP*) trained with 10-fold cross validation on all 11 databases. The model accuracies in predicting the right HA virus class were computed as stated before. In other words, we used 10-fold cross validation of training subsets to compare the prediction accuracy of "neural network with neural network criterion" versus "neural network with AutoMLP criterion" on original dataset (FCdb) as well as 10 datasets trimmed (filtered) via running weighting algorithm by PCA, Uncertainty, Relief, Chi Squared, Gini Index, Deviation, Rule, Gain Ratio, Info Gain, and SVM to find the optimal combination of neural network algorithm with dataset which allows the most accurate prediction (based on 10-fold cross validation).

Neural Net learns a model by means of a feed-forward neural network trained by a back-propagation algorithm (multi-layer perceptron) and the structure of the neural network can be defined by parameter list "hidden_layers". Here a default hidden layer with sigmoid type and size (number of attributes+number of classes)/2+1 created and added to the net. The used activation function is the usual sigmoid function. Therefore, the values ranged of the attributes scaled to -1 and $+1$. The type of the output node was sigmoid because the learning data described a classification task.

AutoMLP algorithm combines ideas from genetic algorithms and stochastic optimization. It maintains a small ensemble of networks that are trained in parallel with different rates and different numbers of hidden units. After a small, fixed number of epochs, the error rate was determined on a validation set and the worst performers were replaced with copies of the best networks, modified to have different numbers of hidden units and learning rates. Hidden unit numbers and learning rates are drawn according to probability distributions derived from successful rates and sizes.

Vi: Statistical Analyses of the Key Distinguishing Protein Attributes of HA Proteins (Selected by Attribute Weighting Algorithms)

Various statistical methods such as descriptive statistics, Univariate Analysis of Variance (ANOVA) and Multivariate Analyses of Variance (MANOVA) were applied to investigated the behaviour and the effects of some important amino acid attributes (selected by attribute weighting algorithms) and some of the important dipeptides (selected by decision tree) models on HA subtypes (H1-H16). The features included: Non-reduced cysteines Ext, Count of Isoleucine, Freq of Cysteine, Freq of Aspartic Acid, Freq of Glutamic Acid (E), Freq of Glutamine, Freq of Arginine, Freq of Tyrosine, Percentage of Histidine, Percentage of Methionin, Percentage of Tryptophan, Percentage of Tyrosine, Count of Phe-Met, Count of Asn-Met, Freq of Pro-Gly, and Freq of Trp-Leu.

Also, the agglomerative hierarchical cluster analysis was applied on dataset of the above mentioned key features consisting of 16 protein attributes per each sequence. These given variables obtained by the feature selection criteria were standardized in order to be equally important in computing distance [99]. The method used in this cluster analysis was single linkage. Manhattan distance was applied to compute the distance among items in this study.

Supporting Information

Figure S1 Clustering of proteins based on the 16 important features found in this study. The key discovered features (obtained by attribute weighting and decision tree models) are highly accurate in prediction and separation of HA subtypes and reinforces the high capability of discovered features as predictors of HA subtyping. The features include: Non-reduced cysteines Ext, Count of Isoleucine, Freq of Cysteine, Freq of Aspartic Acid, Freq of Glutamic Acid, Freq of Glutamine, Freq of Arginine, Freq of Tyrosine, Percentage of Histidine, Percentage of Methionin, Percentage of Tryptophan, Percentage of Tyrosine, Count of Phe-Met, Count of Asn-Met, Freq of Pro-Gly, and Freq of Trp-Leu.
(TIF)

Table S1 The original cleaned protein feature data set for HA sequences (Fcdb). This database contains 7338 protein sequences and 893 protein features. Table S1 is shared by "Googledrive" at the following link: https://drive.google.com/file/d/0B2Npj-saFbgeNjhwRTJubFRJdFk/edit?usp = sharing.
(DOCX)

Table S2 Distribution of important found protein attributes (according to attribute weighting algorithms) in the structure of HA subtypes. The features include:

frequency of Gln, The frequency of Tyr, Percentage of Tyr, Percentage of Cys, The frequency of Glu, Percentage of Try, Count of Ile, The frequency of Arg, Percentage of His, The frequency of Asp, Percentage of Met, Non-reduced Cys extinction coefficient at 280nm, and The frequency of Phe.
(XLSX)

Table S3 Univariate Analysis of Variance (ANOVA) and Multivariate Analyses of Variance (MANOVA) of the effects of important amino acid attributes (selected by attribute weighting algorithms) and some of the important dipeptides (selected by decision tree) models on determining HA subtypes (H1-H16).
(DOCX)

Table S4 Weighting algorithms used for selecting the most important protein features distinguishing different influenza HA subtypes [values (weights) closer to 1 shows higher effectiveness of feature]. Weighting algorithms were weighting by PCA, weighting by Relief, weighting by Uncertainty, weighting by Gini index, weighting by Chi Squared, weighting by Deviation, weighting by Rule, weighting by Correlation, weighting by Gain Ratio, and weighting by Information Gain. Total number of attribute weighting algorithms which have announced an attribute as important (weight >0.5) is also counted.
(XLS)

Acknowledgments

The authors would like to thank Indonesian Research Centre for Veterinary Science (Balitvet) for their assistance. With the special thanks to Dr Simson Tarigan, Risa Indriani, Dr N.L.P.Indi Dharmayanti who provided some parts of the AI sequences for this analysis. The authors greatly appreciate the support from Bioinformatics Research Group, GRC, University of Qom and the School of Molecular & Biomedical Science, University of Adelaide, and School of Animal and Veterinary Science, The University of Adelaide, Australia.

Author Contributions

Conceived and designed the experiments: ME PA NS AT FH EE. Performed the experiments: ME PA NS AT EE. Analyzed the data: ME PA NS AT MA DLA FH EE. Contributed reagents/materials/analysis tools: ME PA NS AT MA DLA FH EE. Wrote the paper: ME PA AT MA DLA FH EE.

References

1. Driskell EA, Pickens JA, Humberd-Smith J, Gordy JT, Bradley KC, et al. (2012) Low Pathogenic Avian Influenza Isolates from Wild Birds Replicate and Transmit via Contact in Ferrets without Prior Adaptation. PLoS ONE 7: e38067.
2. Miotto O, Heiny AT, Albrecht R, García-Sastre A, Tan TW, et al. (2010) Complete-Proteome Mapping of Human Influenza A Adaptive Mutations: Implications for Human Transmissibility of Zoonotic Strains. PLoS ONE 5: e9025.
3. Gao R, Cao B, Hu Y, Feng Z, Wang D, et al. (2013) Human Infection with a Novel Avian-Origin Influenza A (H7N9) Virus. New England Journal of Medicine 368: 1888–1897.
4. Fouchier RAM, Kawaoka Y (2013) Avian flu: Gain-of-function experiments on H7N9. Nature 500: 150–151.
5. Watanabe T, Kiso M, Fukuyama S, Nakajima N, Imai M, et al. (2013) Characterization of H7N9 influenza A viruses isolated from humans. Nature 501: 551–555.
6. Zhou J, Wang D, Gao R, Zhao B, Song J, et al. (2013) Biological features of novel avian influenza A (H7N9) virus. Nature 499: 500–503.
7. Ginsberg J, Mohebbi MH, Patel RS, Brammer L, Smolinski MS, et al. (2009) Detecting influenza epidemics using search engine query data. Nature 457: 1012–1014.
8. Steinhauer DA (2013) Influenza: Pathways to human adaptation. Nature 499: 412–413.
9. Palese P (2004) Influenza: old and new threats. Nat Med 10: S82–87.
10. Pan CE, Wang GP, Liao M, Zhang GH, Jiang SB (2009) High genetic and antigenic similarity between a swine H3N2 influenza A virus and a prior human influenza vaccine virus: A possible immune pressure-driven cross-species transmission. Biochemical and Biophysical Research Communications 385: 402–407.
11. Campitelli L, Donatelli I, Foni E, Castrucci MR, Fabiani C, et al. (1997) Continued evolution of H1N1 and H3N2 influenza viruses in pigs in Italy. Virology 232: 310–318.
12. Adeola OA, Adeniji JA, Olugasa BO (2009) Isolation of influenza A viruses from pigs in Ibadan, Nigeria. Vet Ital 45: 383–390.
13. Scholtissek C, Burger H, Kistner O, Shortridge KF (1985) The Nucleoprotein as a Possible Major Factor in Determining Host Specificity of Influenza H3n2 Viruses. Virology 147: 287–294.
14. Imai M, Kawaoka Y (2012) The role of receptor binding specificity in interspecies transmission of influenza viruses. Current Opinion in Virology 2: 160–167.
15. Rogers GN, Paulson JC, Daniels RS, Skehel JJ, Wilson IA, et al. (1983) Single amino acid substitutions in influenza haemagglutinin change receptor binding specificity. Nature 304: 76–78.
16. Weis W, Brown JH, Cusack S, Paulson JC, Skehel JJ, et al. (1988) Structure of the influenza virus haemagglutinin complexed with its receptor, sialic acid. Nature 333: 426–431.
17. Herfst S, Schrauwen EJA, Linster M, Chutinimitkul S, de Wit E, et al. (2012) Airborne Transmission of Influenza A/H5N1 Virus Between Ferrets. Science 336: 1534–1541.
18. Imai M, Watanabe T, Hatta M, Das SC, Ozawa M, et al. (2012) Experimental adaptation of an influenza H5 HA confers respiratory droplet transmission to a reassortant H5 HA/H1N1 virus in ferrets. Nature 486: 420–428.
19. Russell CA, Fonville JM, Brown AE, Burke DF, Smith DL, et al. (2012) The potential for respiratory droplet-transmissible A/H5N1 influenza virus to evolve in a mammalian host. Science 336: 1541–1547.
20. Shinya K, Hamm S, Hatta M, Ito H, Ito T, et al. (2004) PB2 amino acid at position 627 affects, replicative efficiency, but not cell tropism, of Hong Kong H5N1 influenza A viruses in mice. Virology 320: 258–266.
21. Kumar M, Gromiha MM, Raghava GP (2011) SVM based prediction of RNA-binding proteins using binding residues and evolutionary information. J Mol Recognit 24: 303–313.
22. Xiong W, Guo Y, Li M (2010) Prediction of lipid-binding sites based on support vector machine and position specific scoring matrix. Protein J 29: 427–431.
23. Ebrahimi M, Ebrahimie E (2010) Sequence-based prediction of enzyme thermostability through bioinformatics algorithms. Current Bioinformatics 5: 195–203.
24. Tahrokh E, Ebrahimi M, Ebrahimi M, Zamansani F, Rahpeyma Sarvestani N, et al. (2011) Comparative study of ammonium transporters in different organisms by study of a large number of structural protein features via data mining algorithms. Genes & Genomics 33: 565–575.
25. Lakizadeh A, Agha-Golzadeh P, Ebrahimi M, Ebrahimie E, Ebrahimi M (2011) Engineering Thermostable Enzymes; Application of Unsupervised Clustering Algorithms. Advanced Studies in Biology 3: 63–78.
26. Ebrahimie E, Ebrahimi M, Ebrahimi M (2011) Amino acid features: a missing compartment of prediction of protein function. Natureprecedings.
27. Ashrafi E, Alemzadeh A, Ebrahimi M, Ebrahimie E, Dadkhodaei N (2011) Amino Acid Features of P1B-ATPase Heavy Metal Transporters Enabling Small Numbers of Organisms to Cope with Heavy Metal Pollution. Bioinform Biol Insights 5: 59–82.
28. Ebrahimie E, Ebrahimi M, Sarvestani NR (2011) Protein attributes contribute to halo-stability, bioinformatics approach. Saline Systems 7: 1.
29. Gromiha MM, Suresh MX (2008) Discrimination of mesophilic and thermophilic proteins using machine learning algorithms. Proteins: Structure, Function, and Bioinformatics 70: 1274–1279.
30. Gromiha MM, Yukimitsu Yi (2008) Functional discrimination of membrane proteins using machine learning techniques. BMC Bioinformatics 9: 135.
31. Hosseinzadeh F, Ebrahimi M, Goliaei B, Shamabadi N (2012) Classification of lung cancer tumors based on structural and physicochemical properties of proteins by bioinformatics models. PLoS One 7: e40017.
32. Dugas AF, Jalalpour M, Gel Y, Levin S, Torcaso F, et al. (2013) Influenza Forecasting with Google Flu Trends. PLoS One 8: e56176.
33. Tang ZQ, Lin HH, Zhang HL, Han LY, Chen X, et al. (2009) Prediction of functional class of proteins and peptides irrespective of sequence homology by support vector machines. Bioinform Biol Insights 1: 19–47.
34. Lin HH, Han LY, Zhang HL, Zheng CJ, Xie B, et al. (2006) Prediction of the functional class of lipid binding proteins from sequence-derived properties irrespective of sequence similarity. J Lipid Res 47: 824–831.
35. Hosseinzadeh F, Kayvanjoo AH, Ebrahimi M, Goliaei B (2013) Prediction of lung tumor types based on protein attributes by machine learning algorithms. Springerplus 2: 238.
36. Ong SA, Lin HH, Chen YZ, Li ZR, Cao Z (2007) Efficacy of different protein descriptors in predicting protein functional families. BMC Bioinformatics 8: 300.
37. Ding CH, Dubchak I (2001) Multi-class protein fold recognition using support vector machines and neural networks. Bioinformatics 17: 349–358.

38. Ebrahimi M (2011) Comparing various attributes of prolactin hormones in different species: application of bioinformatics tools. Iranian Journal of Veterinary Research 12: 205–213.
39. Niu XH, Hu XH, Shi F, Xia JB (2012) Predicting protein solubility by the general form of Chou's pseudo amino acid composition: approached from chaos game representation and fractal dimension. Protein Pept Lett 19: 940–948.
40. Tsipouras MG, Tzallas AT, Rigas G, Tsouli S, Fotiadis DI, et al. (2012) An automated methodology for levodopa-induced dyskinesia: assessment based on gyroscope and accelerometer signals. Artif Intell Med 55: 127–135.
41. Macas M, Lhotska L, Bakstein E, Novak D, Wild J, et al. (2012) Wrapper feature selection for small sample size data driven by complete error estimates. Comput Methods Programs Biomed 108: 138–150.
42. Hu X, Sapo M, Nenov V, Barry T, Kim S, et al. (2012) Predictive combinations of monitor alarms preceding in-hospital code blue events. J Biomed Inform 45: 913–921.
43. Huang LT, Gromiha MM (2012) Real value prediction of protein folding rate change upon point mutation. J Comput Aided Mol Des 26: 339–347.
44. Zhao X, Ma Z, Yin M (2012) Using Support Vector Machine and Evolutionary Profiles to Predict Antifreeze Protein Sequences. International Journal of Molecular Sciences 13: 2196–2207.
45. Martinez JG, Carroll RJ, Muller S, Sampson JN, Chatterjee N (2011) Empirical Performance of Cross-Validation With Oracle Methods in a Genomics Context. Am Stat 65: 223–228.
46. McCarthy LC, Newcombe PJ, Whittaker JC, Wurzelmann JI, Fries MA, et al. (2012) Predictive models of choroidal neovascularization and geographic atrophy incidence applied to clinical trial design. Am J Ophthalmol 154: 568–578 e512.
47. Nongpiur ME, Haaland BA, Friedman DS, Perera SA, He M, et al. (2013) Classification algorithms based on anterior segment optical coherence tomography measurements for detection of angle closure. Ophthalmology 120: 48–54.
48. Tamaki T, Yoshimuta J, Kawakami M, Raytchev B, Kaneda K, et al. (2013) Computer-aided colorectal tumor classification in NBI endoscopy using local features. Medical Image Analysis 17: 78–100.
49. Wu W, Dasgupta S, Ramirez EE, Peterson C, Norman JG (2012) Classification Accuracies of Physical Activities Using Smartphone Motion Sensors. J Med Internet Res 14: e130.
50. Xia X-Y, Ge M, Wang Z-X, Pan X-M (2012) Accurate Prediction of Protein Structural Class. PLoS ONE 7: e37653.
51. Quinlan JR (1986) Induction of decision trees. Machine Learning 1: 81–106.
52. Huang L-T, Gromiha MM, Hwang S-F, Ho S-Y (2006) Knowledge acquisition and development of accurate rules for predicting protein stability changes. Computational Biology and Chemistry 30: 408–415.
53. Vapnik V (1995) The Nature of Statistical I. eaming Theory. New York: Spfnger Vef lag.
54. Brown MP, Grundy WN, Lin D, Cristianini N, Sugnet CW, et al. (2000) Knowledge-based analysis of microarray gene expression data by using support vector machines. Proc Natl Acad Sci U S A 97: 262–267.
55. Cui G, Fang C, Han K (2012) Prediction of protein-protein interactions between viruses and human by an SVM model. BMC Bioinformatics 13 Suppl 7: S5.
56. Markowetz F, Edler L, Vingron M (2003) Support vector machines for protein fold class prediction. Biometrical Journal 45: 377–389.
57. Byvatov E, Fechner U, Sadowski J, Schneider G (2003) Comparison of support vector machine and artificial neural network systems for drug/nondrug classification. J Chem Inf Comput Sci 43: 1882–1889.
58. Baseri S, Towhidi M, Ebrahimie E (2011) A Modified Efficient Empirical Bayes Regression Model for Predicting Phenomena with a Large Number of Independent Variables and Fewer Observations; Examples of its Application in Human Disease, Protein Bioinformatics, and Microarray Gene Expression Profiling. Advanced Studies in Biology 3: 181–204.
59. Wen X, Fang J, Diao M, Zhang C (2012) Artificial neural network modeling of dissolved oxygen in the Heihe River, Northwestern China. Environ Monit Assess.
60. Cartwright HM (2008) Artificial neural networks in biology and chemistry: the evolution of a new analytical tool. Methods Mol Biol 458: 1–13.
61. Zhao R, Xu G, Yue B, Liebich HM, Zhang Y (1998) Artificial neural network classification based on capillary electrophoresis of urinary nucleosides for the clinical diagnosis of tumors. J Chromatogr A 828: 489–496.
62. Tsilo LC (2009) Protein secondary structure prediction using neural networks and support vector machines: Rhodes University.
63. Patel AK, Patel S, Naik PK (2012) Prediction and classification of DNA binding proteins into four major classes based on simple sequence derived features using Ann. Digest Journal of Nanomaterials and Biostructures 5: 191–200.
64. Westerlund I, Von Heijne G, Emanuelsson O (2009) LumenP–A neural network predictor for protein localization in the thylakoid lumen. Protein science 12: 2360–2366.
65. He J, Deem MW (2010) Low-dimensional clustering detects incipient dominant influenza strain clusters. Protein Engineering Design and Selection 23: 935–946.
66. Attaluri PK, Zheng X, Chen Z, Lu G (2009) Applying machine learning techniques to classify H1N1 viral strains occurring in 2009 flu pandemic. BIOT-2009: 21.
67. Sherif FF, El Hefnawi M, Kadah Y (2011) Genomic signatures and associative classification of the Hemagglutinin protein for Human versus Avian versus Swine Influenza A viruses. IEEE. pp. 1–8.
68. Ebrahimi M, Ebrahimie E, Shamabadi N, Ebrahimi M (2010) Are there any differences between features of proteins expressed in malignant and benign breast cancers? Journal of Research in Medical Sciences 15: 299–309.
69. Ebrahimie E, Ebrahimi M, Rahpayma M (2010) Investigating protein features contribute to salt stability of halolysin proteins. Journal of Cell and Molecular Research 2: 15–28.
70. Tahrokh E, Ebrahimi M, Ebrahimi M, Zamansani F, Sarvestani NR, et al. (2011) Comparative study of ammonium transporters in different organisms by study of a large number of structural protein features via data mining algorithms. Genes & Genomics 33: 565–575.
71. Ashrafi E, Alemzadeh A, Ebrahimi M, Ebrahimie E, Dadkhodaei N, et al. (2011) Determining specific amino acid features in P1B-ATPase heavy metals transporters which provides a unique ability in small number of organisms to cope with heavy metal pollution Bioinformatics and Biology Insights Accepted.
72. Ebrahimi M, Lakizadeh A, Agha-Golzadeh P, Ebrahimie E (2011) Prediction of thermostability from amino acid attributes by combination of clustering with attribute weighting: a new vista in engineering enzymes. PLoS One 6: e23146.
73. Liu JP (2006) Avian influenza–a pandemic waiting to happen? J Microbiol Immunol Infect 39: 4–10.
74. Zhu H, Webby R, Lam TT, Smith DK, Peiris JS, et al. (2011) History of Swine Influenza Viruses in Asia. Curr Top Microbiol Immunol.
75. Li Z, Chen H, Jiao P, Deng G, Tian G, et al. (2005) Molecular basis of replication of duck H5N1 influenza viruses in a mammalian mouse model. J Virol 79: 12058–12064.
76. Baumgartner C, Lewis GD, Netzer M, Pfeifer B, Gerszten RE (2010) A new data mining approach for profiling and categorizing kinetic patterns of metabolic biomarkers after myocardial injury. Bioinformatics 26: 1745–1751.
77. Bijanzadeh E, Emam Y, Ebrahimie E (2010) Determining the most important features contributing to wheat grain yield using supervised feature selection model. Australian Journal of Crop Science 4: 402–407.
78. Ebrahimi M, Ebrahimie E, Ebrahimi M (2009) Searching for patterns of thermostability in proteins and defining the main features contributing to enzyme thermostability through screening, clustering, and decision tree algorithms. EXCLI Journal 8: 218–233.
79. Sahini L, Tempczyk-Russell A, Agarwal R (2010) Large-scale sequence analysis of hemagglutinin of influenza A virus identifies conserved regions suitable for targeting an anti-viral response. PLoS One 5: e9268.
80. Nunthaboot N, Rungrotmongkol T, Malaisree M, Kaiyawet N, Decha P, et al. (2010) Evolution of human receptor binding affinity of H1N1 hemagglutinins from 1918 to 2009 pandemic influenza A virus. J Chem Inf Model 50: 1410–1417.
81. Wu ZQ, Ji J, Zuo KJ, Xie QM, Li HM, et al. (2010) Cloning and phylogenetic analysis of hemagglutinin gene of H9N2 subtype avian influenza virus from different isolates in China during 2002 to 2009. Poult Sci 89: 1136–1143.
82. Herfst S, Osterhaus ADME, Fouchier RAM (2012) The Future of Research and Publication on Altered H5N1 Viruses. Journal of Infectious Diseases 205: 1628–1631.
83. Radman A, Gredicak M, Kopriva I, Jeric I (2011) Predicting antitumor activity of peptides by consensus of regression models trained on a small data sample. Int J Mol Sci 12: 8415–8430.
84. Tan T, Platel B, Huisman H, Sanchez C, Mus R, et al. (2012) Computer Aided Lesion Diagnosis in Automated 3D Breast Ultrasound Using Coronal Spiculation. IEEE Trans Med Imaging.
85. Li Z, Liu P, Wang W, Xu C (2012) Using support vector machine models for crash injury severity analysis. Accid Anal Prev 45: 478–486.
86. Wang K, Hu X, Wang Z, Yan A (2012) Classification of Acetylcholinesterase Inhibitors and Decoys by a Support Vector Machine. Comb Chem High Throughput Screen.
87. Kuo CC, Lin WS, Dressel CA, Chiu AW (2011) Classification of intended motor movement using surface EEG ensemble empirical mode decomposition. Conf Proc IEEE Eng Med Biol Soc 2011: 6281–6284.
88. Sawagashira T, Hayashi T, Hara T, Katsumata A, Muramatsu C, et al. (2011) An automatic detection method for carotid artery calcifications using top-hat filter on dental panoramic radiographs. Conf Proc IEEE Eng Med Biol Soc 2011: 6208–6211.
89. Collier N, Son NT, Nguyen NM (2011) OMG U got flu? Analysis of shared health messages for bio-surveillance. J Biomed Semantics 2 Suppl 5: S9.
90. Watanabe T, Kawaoka Y (2011) Pathogenesis of the 1918 pandemic influenza virus. PLoS Pathog 7: e1001218.
91. Deng L, Guan J, Dong Q, Zhou S (2009) Prediction of protein-protein interaction sites using an ensemble method. BMC Bioinformatics 10: 426.
92. Ito K (2011) Bioinformatics technologies for the analysis of antigenic evolution of influenza viruses. Uirusu 61: 3–13.
93. Althouse BM, Ng YY, Cummings DA (2011) Prediction of dengue incidence using search query surveillance. PLoS Negl Trop Dis 5: e1258.
94. Wu X, Cai Z, Wan X-F, Hoang T, Goebel R, et al. (2007) Nucleotide composition string selection in HIV-1 subtyping using whole genomes. Bioinformatics 23: 1744–1752.
95. Qi J, Wang B, Hao B-I (2004) Whole Proteome Prokaryote Phylogeny Without Sequence Alignment: A K-String Composition Approach. Journal of Molecular Evolution 58: 1–11.
96. Qi J, Hao B (2004) Prokarote phylogeny without sequence allignment: from advoidance signature to composition distance. Journal of Bioinformatics and Computational Biology 02: 1–19.

97. Hemmatzadeh F, Sumarningsih S, Tarigan S, Indriani R, Dharmayanti NL, et al. (2013) Recombinant M2e Protein-Based ELISA: A Novel and Inexpensive Approach for Differentiating Avian Influenza Infected Chickens from Vaccinated Ones. PLoS One 8: e56801.
98. Zhao Y, Zhang Y (2008) Comparison of decision tree methods for finding active objects. Advances in Space Research 41: 1955–1959.
99. Manly BFJ (1994) Multivariate Statistical Methods: A Primer, Second Edition: Chapman & Hall.
